# Predicting Impacts of Future Climate Change on the Distribution of the Widespread Conifer *Platycladus orientalis*

**DOI:** 10.1371/journal.pone.0132326

**Published:** 2015-07-01

**Authors:** Xian-Ge Hu, Yuqing Jin, Xiao-Ru Wang, Jian-Feng Mao, Yue Li

**Affiliations:** 1 National Engineering Laboratory for Tree Breeding, College of Biological Sciences and Technology, Beijing Forestry University, Beijing, China; 2 Key Laboratory of Genetics and Breeding in Forest Trees and Ornamental Plants, Ministry of Education, College of Biological Sciences and Technology, Beijing Forestry University, Beijing, China; 3 Department of Ecology and Environmental Science, Umeå University, Umeå, Sweden; Nanjing Forestry University, CHINA

## Abstract

Chinese thuja (*Platycladus orientalis*) has a wide but fragmented distribution in China. It is an important conifer tree in reforestation and plays important roles in ecological restoration in the arid mountains of northern China. Based on high-resolution environmental data for current and future scenarios, we modeled the present and future suitable habitat for *P*. *orientalis*, evaluated the importance of environmental factors in shaping the species´ distribution, and identified regions of high risk under climate change scenarios. The niche models showed that *P*. *orientalis* has suitable habitat of ca. 4.2×10^6^ km^2^ across most of eastern China and identified annual temperature, monthly minimum and maximum ultraviolet-B radiation and wet-day frequency as the critical factors shaping habitat availability for *P*. *orientalis*. Under the low concentration greenhouse gas emissions scenario, the range of the species may increase as global warming intensifies; however, under the higher concentrations of emissions scenario, we predicted a slight expansion followed by contraction in distribution. Overall, the range shift to higher latitudes and elevations would become gradually more significant. The information gained from this study should be an useful reference for implementing long-term conservation and management strategies for the species.

## Introduction

The Fifth Assessment Report (AR5) of the Intergovernmental Panel on Climate Change (IPCC) [1] predicts that global climate warming will continue and the average temperature on the planet will have increased by 0.3–4.5°C through the end of the 21st century (relative to the 1986–2005 baseline). Current changes have already markedly affected the temporal and spatial properties of climate in China: (1) the spatial and temporal distributions of the heat resource have become very uneven, (2) the cold regions of the north have experienced rapid increases in temperature, and (3) the spatial unevenness of precipitation is increasing [2]. Changes in the spatiotemporal pattern of climate will have strong impacts on soil properties, plant phenology, plant diseases, insect pests, and the properties of forest ecosystems [1].

Climate shifts alter species’ geographic distributions, which in turn affect the patterns of climate change since surface vegetation has a major controlling effect on atmospheric properties. Consequently, a capacity for predicting the distribution patterns of species under future climate conditions will contribute substantially to the academic disciplines of biogeography and global change biology. Predicting the future impact of climate change on the areal extent of landscape containing appropriate habitat for particular species [3–5] will alert managers to the potential threats of climate change on species distribution ranges and effectively guide the establishment of biological strategies for resource development and utilization, including germplasm preparation and storage. Predictions on critical habitat availability will also contribute to better understanding ecological system stability and the distribution patterns of genetic variation within species [6].

Species distribution modeling (SDM) is widely used in ecology, biogeography, and evolutionary studies [7]. In recent years, SDM using Maxent method has contributed significantly to the prediction of biodiversity loss under future climate scenarios [8] and to the rational use of environmental resources [9] threatened by upcoming climate changes. Importantly, Maxent is able to function with presence-only data, such as those of species’ distribution records. It requires only geo-referenced occurrence data to predict the distribution of suitable habitat for a species through determinations of the probability distribution of maximum entropy [10] coupled with environmental information for the whole study area.

*Platycladus orientalis* (L.) Franco, a member of the family Cupressaceae, is a widespread conifer in China [11]. This species is able to endure drought and persist on barren soil, and consequently, it is commonly used for ecological restoration projects in arid mountain landscapes of northern China, where the species is used in protection forests for sand stabilization and soil erosion control. The species has unique abilities in the absorption and accumulation of atmospheric pollutants (SO_2_, Cl_2_) and heavy metal pollutants in soil (Cu, Zn, among others) [12]. Its wood is strong and decay-resistant, making it valuable in building, furniture, and ship construction [13]. Until now, little is known about the properties of the habitat distribution for this tree, and the important eco-environmental factors shaping the suitability. Whether changing climate will impact the areal extent of suitable habitat for *P*. *orientalis* is a crucial issue given the ecological and economic significance of the species, and enhancing the powers of prediction would contribute to management planning.

Based on an extensive collection of geo-referenced occurrence records for *P*. *orientalis* and large-scale, high-precision meteorological environmental data, this study aims (1) to identify the main impact factors for habitat suitability shift of *P*. *orientalis*, (2) to examine the properties and trends of shifts in the areal extent of suitable habitat under a variety of current and future climate scenarios, and (3) to project and quantify the spatial pattern of shifts in the areal extent of suitable habitat under future climate conditions, thereby providing a theoretical reference framework for global change biology, as well as for the plantation, management, and utilization of *P*. *orientalis*.

## Materials and Methods

### The occurrence and distribution of *P*. *orientalis*

To obtain general information relating to the occurrence of *P*. *orientalis* across its whole range, we first accessed distributions reported in the literature [14–16] and the China Virtual Herbarium (CVH, http://v5.cvh.org.cn/) database, which holds plant distribution records from all main herbaria across the country. In total, we collected 691 unrepeatable geo-referenced occurrence records (604 occurrence points from the CVH and 87 occurrence points from literatures, shown in Supporting Information S1 Dataset). When occurrence records lacked exact geo-coordinates, we used Google Earth (http://ditu.google.cn/) to determine the latitude and longitude.

### Current environmental parameters

The distribution area of *P*. *orientalis* is affected by meteorological factors, such as temperature, moisture, light, soil conditions and landscape properties. On the basis of earlier screenings of related variables [17], we selected eight meteorological and 10 environmental parameters that are closely related to plant growth (Table 1).

**Table 1:** Environmental parameters used to predict the potential geographic distribution of *Platycladus orientalis*.

Code	Name	Resolution	Unit	Source
Bio1	Annual mean air temperature	30″×30″	°C×10	http://www.worldclim.org/
Bio2	Mean diurnal air temperature range	30″×30″	°C×10	http://www.worldclim.org/
Bio3	Isothermality	30″×30″	×100	http://www.worldclim.org/
Bio4	Air temperature seasonality	30″×30″	×100	http://www.worldclim.org/
Bio5	Max air temperature of the warmest month	30″×30″	°C×10	http://www.worldclim.org/
Bio12	Annual precipitation	30″×30″	mm	http://www.worldclim.org/
Bio14	Precipitation of the driest month	30″×30″	mm	http://www.worldclim.org/
Bio15	Precipitation seasonality (coefficient of variation)	30″×30″	mm	http://www.worldclim.org/
FRS	Ground-frost frequency	30″×30″		http://www.ipcc-data.org/obs/cru_ts2_1.html
GDD	Growing degree days	0.5°×0.5°	day	http://www.sage.wisc.edu/atlas/index.php
SC	Soil organic carbon	0.5°×0.5°		http://www.sage.wisc.edu/atlas/index.php
SpH	Soil pH	0.5°×0.5°		http://www.sage.wisc.edu/atlas/index.php
VAP	Vapor pressure	0.5°×0.5°	hPa	http://www.ipcc-data.org/obs/cru_ts2_1.html
WET	Wet-day frequency	0.5°×0.5°		http://www.ipcc-data.org/obs/cru_ts2_1.html
UVB1	Annual mean UV-B	30″×30″	J m^-2^ •day^-1^	http://www.ufz.de/gluv/
UVB2	UV-B seasonality	30″×30″	J m^-2^ •day^-1^	http://www.ufz.de/gluv/
UVB3	mean UV-B of lightest month	30″×30″	J m^-2^ •day^-1^	http://www.ufz.de/gluv/
UVB4	mean UV-B of lowest month	30″×30″	J m^-2^ •day^-1^	http://www.ufz.de/gluv/

The eight bioclimatic layers (Bio1, Bio2, Bio3, Bio4, Bio5, Bio12, Bio14, Bio15) included in our modeling exercise were obtained from Worldclim (http://www.worldclim.org/) [18]. Climate and environmental data were obtained as follows: soil organic carbon (SC), soil pH (SpH), and growing degree days (GDD) from the Center for Sustainability and the Global Environment (http://www.sage.wisc.edu/atlas/index.php); ground-frost frequency (FRS), wet-day frequency (WET), and vapor pressure (VAP) from the IPCC database (http://www.ipcc-data.org/obs/cru_ts2_1.html); and global UV-B radiation (UVB1, UVB2, UVB3, UVB4) from the gIUV database (http://www.ufz.de/gluv/) [19]. These data were preprocessed to a general spatial resolution of 30′′ latitude/longitude (ca. 1 km^2^ at ground level).

### Future climate scenarios and data

To support the IPCC, the international climate research community has participated in a comparison project titled the “Coupled Model Intercomparison Project 5 (CMIP5)” [1]. Within this framework, coordinated experiments using climate and earth system models have been organized to research and answer questions on the mechanisms and properties of climate change. The Fifth IPCC Assessment Report (AR5) was published on September 30, 2013 [1]. It defined a new set of scenarios to take account of the rising complexity of model calculations and to analyze the effects of different political measures. The Representative Concentration Pathways (RCPs) represent the full bandwidth of possible future emission trajectories. Four RCPs (i.e., RCP2.6, RCP4.5, RCP6.0, and RCP8.5) were coded according to a possible range of radiative forcing values in the year 2100 relative to preindustrial values (+2.6, +4.5, +6.0, and +8.5 W•m^-2^, respectively) [20]. The extreme weather variations will pose greater influence to geographical distribution pattern and its temporal and spatial variation of plant. So we selected climate variables for simulation in four climate change scenario/year combinations: RCP2.6–2050 (average for the years 2041–2060 under scenario RCP2.6), RCP2.6–2070 (average for the years 2061–2080 under the scenario RCP2.6), RCP8.5–2050 (average for the years 2061–2080 under the scenario RCP8.5), and RCP8.5–2070 (average for the years 2061–2080 under the scenario RCP8.5), these data all with better representation. The CMIP5-derived future climate scenario data that we used were coded (as BCC-CSM1-1) and generated by the Beijing Climate Center (http://cmdp.ncc.cma.gov.cn/en/). Our four selected future climate data sets were downloaded from the World Climate Database (http://www.worldclim.org/) [18]. Currently only meteorological data (we selected 8 in our study) under future scenario are available for related studies. The 10 environmental parameters (SC, SpH, GDD, FRS, WET, VAP and 4 UV-B radiation parameters) were remained unchanged for the following analyses of SDM projection under future climate conditions.

### Current and future potential habitat predictions

Using data for the 18 environmental parameters and the occurrence of *P*. *orientalis*, we performed SDM for current climate conditions using the maximum entropy approach in Maxent v3.3.3 software operating with default settings [10]. Model predictions were then checked against real observations using the area under the curve (AUC) of a receiver operating characteristic (ROC) plot [21]. A ROC curve shows the performance of a model whose output depends on a threshold parameter, it tests whether a model classifies species presence more accurately than random predictions. A perfect model has an AUC of 1, but performance is good when the AUC is > 0.9 [22]. Conversion of the continuous suitability index maps to binary habitat and non-habitat charts required a probability threshold to determine potential changes in future habitat of *P*. *orientalis*. To define habitat and non-habitat for *P*. *orientalis*, we used the “maximum training sensitivity plus specificity” threshold which has been shown to produce highly accurate predictions [23].

Using Maxent-generated response curves, we obtained relationships between habitat suitability for *P*. *orientalis* and environmental variables. Based on predictor contributions in the Maxent model, we were able to identify the main impact factors for habitat suitability in *P*. *orientalis*.

After modeling the current suitable habitat area for *P*. *orientalis* with current climate data, we performed modeling projections for future climate scenarios (RCP2.6–2050, RCP2.6–2070, RCP8.5–2050, and RCP8.5–2070) to predict the future suitable habitat area for the species. The SDM projections were calculated with Maxent software. We cross-checked future suitable habitat areas against the current distribution area of *P*. *orientalis* to identify regions that had become (i) unsuitable, (ii) suitable, and (iii) remained unchanged with respect to suitability; we then calculated and illustrated the areas of regions identified in (i)-(iii).

### The core distributional shifts

To further examine the trend of suitable area changes, we also calculated and compared the centroids of future and current suitable areas by using a python-based GIS toolkit, SDMtoolbox [24]. The SDMtoolbox calculates the distributional changes between two binary SDMs (e.g. current and future SDMs) [24], and this analysis is focused on summarizing the core distributional shifts of the ranges of *P*. *orientalis*. This analyses reduces species' distribution to a single central point (known as a centroid) and creates a vector file depicting magnitude and direction of predicted change through time. We examined the distributional shifts by tracking the changes in centroid changes among different SDMs.

## Results

### The species distribution model and its accuracy

**Fig 1:**
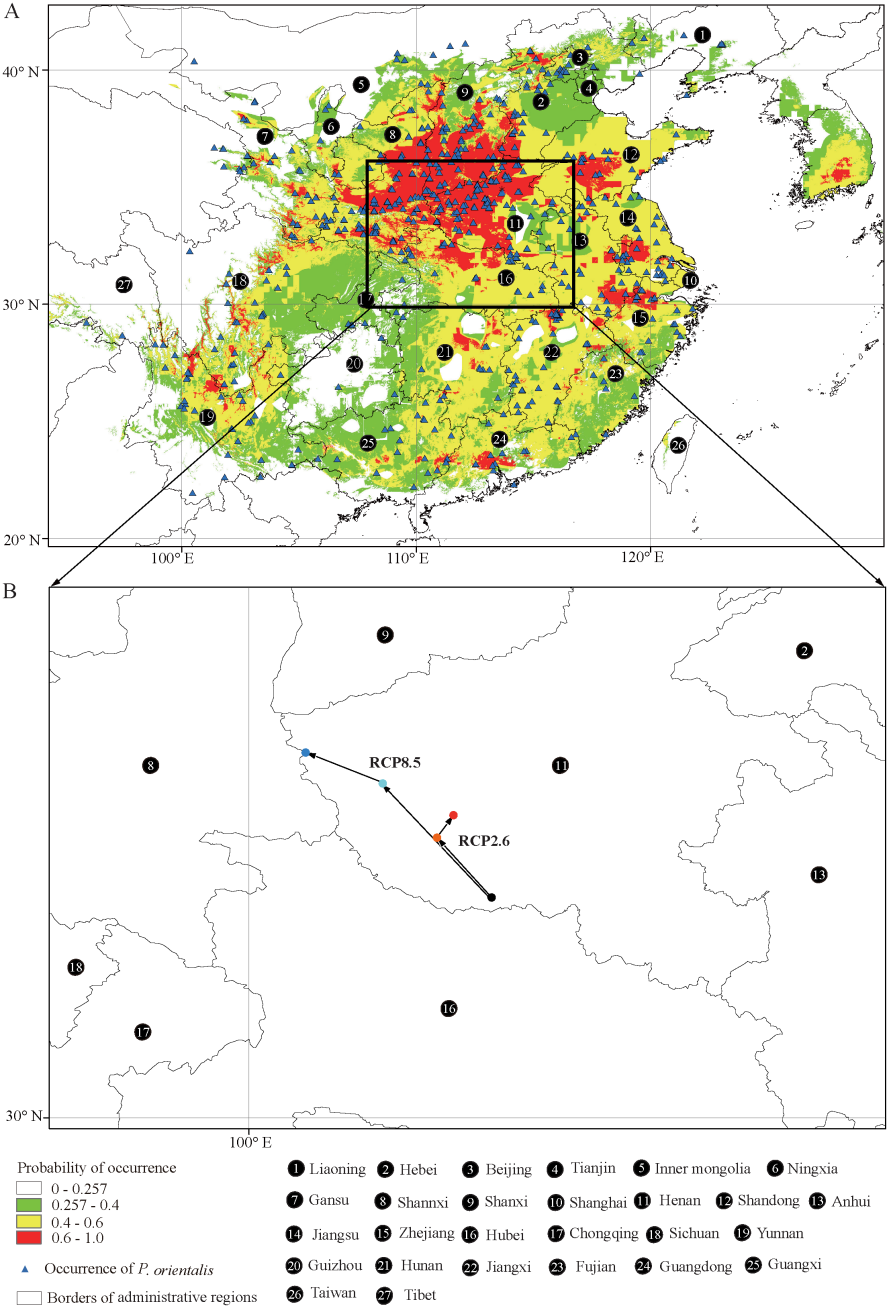
Current species distribution model and the core distributional shifts under different climate scenario/year for *Platycladus orientalis*. A, The occurrence and current species distribution model (SDM) for *Platycladus orientalis*. B, the core distributional shifts under different climate scenario/year combination. Black dot indicates the geometric center of suitable area under current climate condition; orange dot and red dot indicate the geometric centers of future suitable areas of 2050 and 2070 under the climate scenario of RCP2.6; wathet dot and blue dots indicate the geometric centers of future suitable areas of 2050 and 2070 under the climate scenario of RCP8.5, and the arrows depicting magnitude and direction of predicted change through time.

Based on known occurrences of *P*. *orientalis* and current climate data, we generated geographic distribution maps predicting areas where *P*. *orientalis* might occur. The model performance for *P*. *orientalis* was better than random (AUC = 0.952); thus, the model performed well in predicting the suitable habitat area for the species. Our “maximum training sensitivity plus specificity” threshold value of 0.257 was obtained from the 10th percentile training presence occurrences of the species. The suitable habitat areas for *P*. *orientalis* were divided by these calculations into three categories: (i) 0.257–0.400, which included low habitat suitability areas that we color-coded green; (ii) 0.400–0.600, which included areas of moderate habitat suitability that we color-coded yellow; and (iii) 0.600–1.000, which included highly habitat suitable areas that we color-coded red. We constructed geographic distribution maps predicting areas where *P*. *orientalis* might occur (Fig 1A). Currently suitable habitat areas for *P*. *orientalis* were predicted to occur in the southern part of Inner Mongolia, Jilin, Liaoning, eastern Gansu, eastern Sichuan, north–central Yunnan, northern Guangdong, and Guangxi. The predicted areas of suitable habitat for *P*. *orientalis* were continuous and spanned the region from northern to southern sectors of China. The current areas suitable for *P*. *orientalis* were predicted to encompass ca. 4.2×10^6^ km^2^.

### Important environmental variables

**Fig 2:**
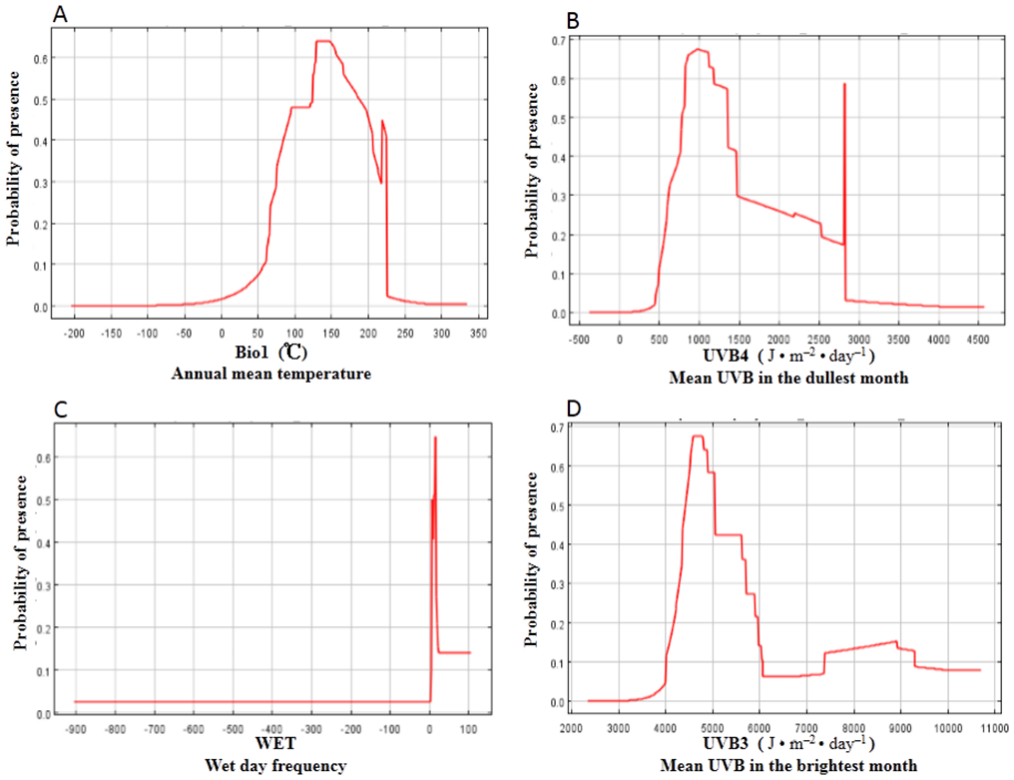
Response curves for important environmental predictors in the species distribution model for *Platycladus orientalis*.

Among the 18 environmental variables annual mean temperature (Bio1), mean UV-B in the dullest month (UVB4), wet-day frequency (WET), and mean UV-B in the brightest month (UVB3) made the greatest contributions to the distribution model for *P*. *orientalis* relative to other variables. Annual mean temperature made the largest contribution (28.9%); the contributions of mean UV-B in the dullest month, wet-day frequency, and mean UV-B in the brightest month were 21.4%, 12.8%, and 10%, respectively. The cumulative contributions of these factors reached values as high as 73.1%. Thus, these four parameters were the major environmental variables we used for constructing the predicted distribution area of *P*. *orientalis*. Using the response curve (Fig 2), we obtained the thresholds (existence probability > 0.257) for the main bioclimatic parameters: mean annual temperature (Bio1) ranged from 7.5 to 25°C, mean UV-B in the dullest month (UVB4) ranged from 600 to 1490 J•m^-2^•day^-1^, wet-day frequency (WET) ranged from 1% to 10%, and mean UV-B in the brightest month (UVB3) ranged from 4200 to 5800 J•m^-2^•day^-1^.

### Future changes in suitable habitat area

**Fig 3:**
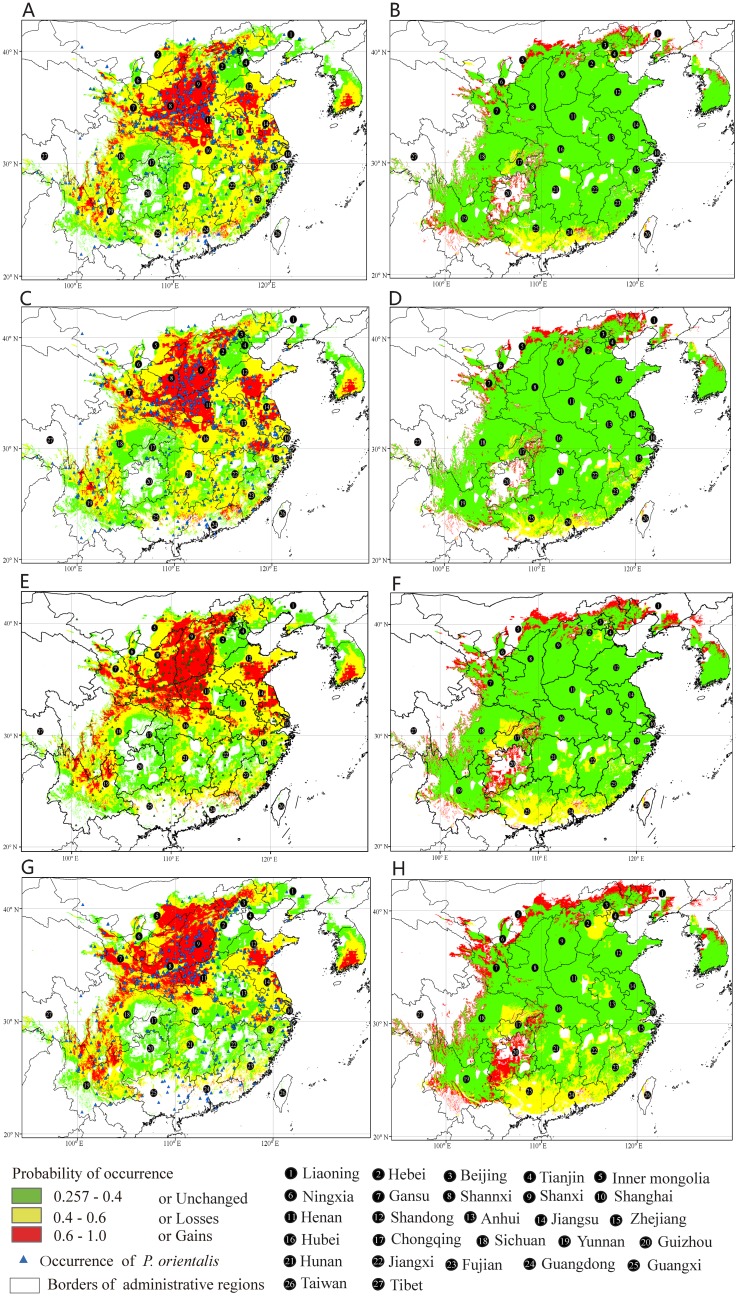
Future species distribution models (SDMs) and their spatial shifts for *Platycladus orientalis* under climate change scenarios RCP2.6 and RCP8.5. A, SDM for *P*. *orientalis* under future climate scenario RCP2.6 in the year 2050. B, SDM for *P*. *orientalis* under future climate scenario RCP2.6 in 2070. C, SDM for *P*. *orientalis* under future climate scenario RCP8.5 in 2050. D, SDM for *P*. *orientalis* under future climate scenario RCP8.5 in 2070. E, Comparison between the current SDM and the SDM under future climate scenario RCP2.6 in the year 2050. F, Comparison between the current SDM and the SDM under future climate scenario RCP2.6 in 2070. G, Comparison between the current SDM and the SDM under future climate scenario RCP8.5 in 2050. H, Comparison between the current SDM and the SDM under future climate scenario RCP8.5 in 2070.

Maxent predicted gains in suitable habitat area for the future climate scenario/year combination RCP2.6–2050 in the southern sector of Inner Mongolia and Liaoning, northern Hebei, central of Yunnan, and Guizhou (Fig 3A and 3B). The predicted area gain amounted to 0.35×10^6^ km^2^, which was 8.35% of the currently suitable habitat area. Expansions in area increased with increasing latitude and elevation (Table 2). The losses of suitable habitat area of ca. 0.26×10^6^ km^2^ (6.21% of the currently suitable habitat area), were predicted for the southern sectors of Guangdong Province and the Guangxi Autonomous Region, and the western part of Chongqing. The areas with a high risk of habitat loss were mostly concentrated at low latitudes. Of the current area with suitable habitat, 93.79% remained unchanged (ca. 3.96×10^6^ km^2^) for the future climate scenario/year combination RCP2.6–2050. The total suitable habitat area for *P*. *orientalis* increased by ca. 0.096×10^6^ km^2^ (2.15% of the total). Overall, we saw a restricted range expansion of suitable habitat area under climate scenario (RCP2.6) (Fig 3A and 3B, Table 2).

**Table 2:** Dynamics of changes in suitable habitat area for *Platycladus orientalis* under four combinations of future climate scenario/year.

Future climate scenario/year combination	Area (×10^6^ km^2^)					Proportion of area (%)			
	Future	Loss	Gain	Unchanged	Total^1^	Loss	Gain	Unchanged	Total^2^
RCP2.6–2050	4.31	0.26	0.35	3.96	0.096	6.21	8.35	93.79	2.15
RCP2.6–2070	4.27	0.29	0.34	3.93	0.054	6.77	8.03	93.23	1.26
RCP8.5–2050	4.27	0.48	0.52	3.75	0.037	11.39	12.26	88.61	0.87
RCP8.5–2070	4.08	0.82	0.68	3.4	-0.14	19.43	16.08	80.57	-3.36

^1,2^Negative values indicate suitable habitat area contractions.

Under the future climate scenario of RCP2.6–2070, Maxent predicted gains in suitable habitat area in the southern sectors of Inner Mongolia, Liaoning, the northern sector of Hebei, and the central and southern sectors of Guangxi and Guizhou (Fig 3C and 3D, Table 2). The areal extent of gains amounted to 0.34×10^6^ km^2^ (8.03% of the currently suitable habitat area). Expansions toward higher latitude and elevations were more marked than for the RCP2.6–2050 climate scenario/year combination. Losses of suitable habitat area were mostly concentrated in the central and southern sectors of Guangdong and Guangxi, western Chongqing, northeastern Guangxi, northeastern and central Fujian, and southern Zhejiang; losses amounted to ca. 0.29×10^6^ km^2^ (6.77%) and were mostly concentrated in low latitudes. In addition to overall losses, the predicted suitable habitat area became fragmented and discontinuous in southern China, while suitable habitat expansions were predicted toward northern areas. Areas remaining unchanged in the projection amounted to ca. 3.93×10^6^ km^2^ (93.23%). The total suitable habitat area expanded by ca. 0.05×10^6^ km^2^ (1.26%), but the gains were smaller than the projection for future climate scenario/year combination RCP2.6–2050 (Fig 3A and 3B, Table 2).

Maxent predicted gains in suitable habitat area in very limited regions of northern Shanxi and the central sector of Guangxi under the future climate scenario/year combination RCP8.5–2050 (Fig 3E and 3F). These gains of ca. 0.52×10^6^ km^2^ (11.26% of the current suitable habitat area) were located at high latitudes. The shifts in suitable area got more and more toward higher latitudes and elevations and also expansion to the Yunnan-Guizhou plateau. Losses of suitable habitat area were located mainly in the central and southern sectors of Guangxi and Guangdong, eastern Sichuan, western Chongqing, eastern Hebei, and sectors of Hunan, Jiangxi, and Fujian, and amounted to 0.48×10^6^ km^2^ (11.39%). There were 88.61% of the currently suitable habitat area remained unchanged (ca. 3.75×10^6^ km^2^). The total suitable area was expanded by ca. 0.036×10^6^ km^2^ (0.87%).

Under RCP8.5–2070, Maxent predicted gains in suitable habitat area in the southern sectors of Inner Mongolia and Liaoning, northern sectors of Shanxi and Hebei, and central and southern sectors of Guangxi and Guizhou, amounting to ca. 0.68×10^6^ km^2^ (16.08% of the currently suitable habitat area). The gains toward higher latitudes and elevations, and the expansion to the Yunnan-Guizhou plateau, were very obvious. The losses of suitable habitat area were located mainly in the central and southern sectors of Guangxi and Guangdong, eastern Sichuan, western Chongqing, eastern Hebei, and sections of Hunan, Jiangxi, and Fujian, and amounted to 0.82×10^6^ km^2^ (19.43%). Most of the losses were located at low latitudes. A trend of fragmentation and northerly migration was also apparent. Approximately 3.40×10^6^ km^2^ of suitable habitat area (80.57%) remained unchanged. The total suitable habitat area was reduced by ca. 0.14×10^6^ km^2^ (3.36%), suggesting that under the future climate scenario/year combination RCP8.5–2070, high concentrations of greenhouse gases would substantially degrade habitat for *P*. *orientalis* (Fig 3G and 3H, Table 2).

### The core distributional shifts

The centroid of the current habitat was located at the position of 112.75E in longitude and 32.52N in latitude in south of Henan province (Fig 1B). The centroid of future suitable area shifted to the position (112.12E, 33.26N) in the northwest under RCP2.6–2050, to the position (112.33E, 33.46N) under RCP2.6–2070. Under RCP8.5–2050, the centroid of future suitable area was located at a more northwest position (111.51E, 33.83N), and even more northwest at the position (110.64E, 34.18N) under RCP8.5–2070. Overall, we saw the core distributional shift expressed a northwest tendency under both future emission trajectories (RCP2.6, RCP8.5), and a relatively weak strength was found under the emission trajectory of lower representative concentration, RCP2.6 (Fig 1B).

## Discussion

Climate modeling of tree species distributions has demonstrated that future global climate change will have significant impacts on forest ecosystems [25–26]. Discrepancies do occur between different climate modeling systems [27], but the approach nevertheless functions as an important research tool for assessing and predicting future changes in species distribution [28]. Despite the great strides in climate research made in recent years, most studies on plant species in China provide only approximate locations of change in habitat and rough descriptions of broad trends [29–31]. In the present study, we performed a more detailed study on the suitable habitat of *P*. *orientalis* that will function as an important first step in developing strategies and policies for management and utilization of this important forest tree.

*P*. *orientalis* has a wide but fragmented distribution in China. It ranges across the southern sector of Inner Mongolia, Jilin and Liaoning, northern Shanxi, Gansu, Sichuan, Guizhou, Yunnan, Guangdong, and northern Guangxi [32]. It also occurs in Japan and North Korea. Based on large-scale environmental data, our modeling study projected a distribution of ca. 4.2×10^6^ km^2^ for the species under current climate conditions, in which the center areas are in mid-western Henan, the central and southern sectors of Shanxi and Shaanxi, eastern Gansu, northern Hubei, southwestern Jiangsu, central Shandong, southeastern Anhui, central Guangdong, and northern Yunnan. This model is in agreement with a previous study that located the most suitable areas of the species in typical sub-arid regions in western Henan, Shanxi, Shaanxi, and Gansu [33], but the potential range we identified was broader.

In our modeling exercise, temperature and wetness are among the most important factors affecting the distribution of *P*. *orientalis*. Previous studies have shown that seeds germination rate of *P*. *orientalis* decreases rapidly with decreasing of matric water potential [34]. Water availability is correlated with many environmental factors that influence the physiological and biochemical processes of plants, e.g. soil moisture is shown to be the main factor affecting the plant assimilation rate [35]. The number of branches and female flowers of *P*. *orientalis* are significantly associated with the average annual rainfall [36] while the male flower development are closely related to both temperature and precipitation [37]. On the other hand, the seedling emergence rate and death rate of *P*. *orientalis* are directly affected by temperature [36]. All these hydrothermal factors may have played main roles in shaping the ecological adaptation of *P*. *orientalis*, and have great impacts on the distribution of this conifer tree.

The intensity of UV radiation may also have a major role in shaping the distribution range of *P*. *orientalis*. UV-B reaching the ground has a great impact on the subaerial organs of plants [38]. It affects plant morphology by inhibiting stem elongation and leaf area expansion in many species subjected to elevated UV-B [39]. UV-B radiation decreases photosynthetic activities [40] and influences the functioning of plant protective mechanisms [41]. This form of radiation interacts with other environmental factors, such as water stress, increased CO_2_ concentration, and the availability of nutrients [42]. Levels of these factors modulate the effects of UV-B, which in turn may affect plant responses to other environmental parameters [43–45]. Previous studies have shown that the light saturation point, net photosynthesis, photorespiration of *P*. *orientalis* are closely related to the component of light source [34]. However, little is currently known about the specific effects of UV-B radiation on the changes in the physiology biochemistry of *P*. *orientalis*, and we recommend quantitative studies in the future.

Mountain forest ecosystems in the Northern Hemisphere forest are likely to shift to higher elevations as the atmosphere warms [46–48]. Consistently, our present study predicted upward shifts (Figs 1B and 3). It was predicted that under the low concentration greenhouse gas emissions scenario (RCP2.6), suitable habitat range will increase as global warming intensity proceeds. Under the higher concentrations of the high greenhouse gas emissions scenario (RCP8.5), we predicted a slight expansion in the next 40 years around 2050 followed by the slight contraction in the next 70 years around 2070. Overall, our prediction showed that the shift in distribution of suitable habitat areas to high latitudes and elevations would become gradually more significant, while low-latitude regions, such as Guangdong and Guangxi, would see a significant decrease in distribution. Our study emphasizes that forest surveillance should be planed for marginal populations both in low and high latitudes, so as to give a reasonable management advice in context of future climate change.

Although species are generally migrating to higher latitudes and elevations as climates warm [46, 49], the trends may differ between widely distributed species and those that have narrow ranges of occurrence. Plants with small distribution usually have a constrained ecological adaptability and are more susceptible to the impact of climate change. As an example, the suitable habitat area of the herb, *Sinopodophyllum hexandrum*, was predicted to fluctuate widely under future climate changes [29]. Plants with larger distribution may have broader adaptability and greater resistance to climate change. We found, under the future climate conditions, the distribution of *P*. *orientalis* increased slightly at first, but then began to decrease and fragment as the suitable areas shifted to higher latitudes and elevations during climate warming intensification; these trends are in line with those of previous studies [31, 50–52]. For example, the suitable habitat area for *Pinus massoniana* will shrink [53–54] and that of *Pseudotaxus chienii* will likely become fragmented [55] and reduced under climate change. Habitat fragmentation represents a major threat to tree populations by reducing local population size and gene flow from other populations, which in turn can decrease outcrossing rates, genetic variation [56–59] and the adaptation to future climates. Little is known about the magnitude, direction or time scale of habitat fragmentation impacts on reproductive success and ultimately population viability of *P*. *orientalis*. In addition to shifts to higher latitudes and elevations, attentions should also be taken to fragmentation and shrinkage in habitats in evaluating the climate change impacts and developing a global change forest management strategy.

The combined effects of climatic changes and future land use transformations will increase unsuitable habitats more than one can predict [60]. Our analysis suggested that *P*. *orientalis* has a broad adaptability under the current and future climates, but much of the suitable area might disappear due to land use transformations and humans exploitation for economic purpose. Practically the entire suitable habitat of *P*. *orientalis* is transected by or within the vicinity of populated places, and much of the suitable area for *P*. *orientalis* has already been converted into agriculture or urbanized. Studies are still needed to qualify the anthropogenic impacts on the adaptation to future climates for this wide-spread conifer tree.

Seven sectors of high risk in the face of climate change have been identified in China: (i) northern semiarid, semihumid areas; (ii) the northwestern semiarid region; (iii) the North China Plain; (iv) Southern Hills; (v) the southwestern mountain region; (vi) the southwestern limestone mountain region; and (vii) the Qinghai-Tibet Plateau [61]. Areas that may become unsuitable for *P*. *orientalis* under the future climate scenario/year combination RCP2.6–2050 matched susceptible regions previously identified. Under combination RCP2.6–2070, regions losing the most suitable habitat area were the susceptible sectors previously identified as (iv) and (vi) above, and the South Hills area. Large losses of suitable habitat also occurred in sectors (iv) and (vi) under scenario/year combination RCP8.5–2050 and RCP8.5–2070. The predictions of distribution change in the present study were performed based on two extreme greenhouse emission scenarios (RCP2.6 and RCP8.5) from one General Circulation Model (GCM). The projections for future climate can be very different among GCMs. Nonetheless, we hope the extreme scenarios we molded will present a broadly forecast for the species´ range shift under future conditions. More modeling under a range of climate conditions are needed to reach a better resolution and a stronger recommendation for conservation operations.

Adaptation strategies to climate change, such as assisted migration, have gained increasing attention as we seek to help plants keep pace with changing climatic conditions [62]. Our modeling predicted an apparent shift of suitable habitat area for *P*. *orientalis* toward high latitudes and elevations, and also identified the low elevation regions in southern China facing high risks of habitat loss. We propose the following management strategy in response to future climate change: (1) implementation of seed collection and banking actions across the entire species range to provide a safety net in the face of natural and human uncertainty; (2) a focus on target populations likely to go extinct under climate change, especially those at low altitudes and latitudes; and (3) the use of seeds collected in the southern portion of the range to augment extant plantation in the northern portion, thereby introgressing potentially adaptive traits.

## Conclusions

Based on Maxent modeling, we identified a broad habitat for *P*. *orientalis* under current climate conditions. The projected distribution matches well the extant range of the species across most of eastern part of China. Annual temperature, monthly minimum ultraviolet-B radiation (UV-B), wet-day frequency, and monthly maximum UV-B were important factors shaping the habitat availability for this species. Our model predicted that the shift of habitat to higher latitudes and elevations will become gradually more significant during climate warming intensification, while the species abundance in low-latitude regions will decrease. The projected spatial and temporal pattern of range shifts for *P*. *orientalis* will be a useful reference in developing forest management and conservation strategies for this ecologically important species.

## Supporting Information

S1 Dataset Occurrence records of *P*. *orientalis* used for ecological modeling. (CSV)
(CSV)
